# A Rare Case of Hepatic Gastrinoma

**DOI:** 10.7759/cureus.43383

**Published:** 2023-08-12

**Authors:** Eli A Zaher, Shayet Hossain Eshan, Parth Patel, Zahin Islam Rafa, Daria Zaher

**Affiliations:** 1 Department of Internal Medicine, Ascension Saint Joseph Hospital, Chicago, USA; 2 Department of Internal Medicine, Ibn Sina Medical College Hospital, Dhaka, BGD; 3 Department of Internal Medicine, University Clinical Hospital in Bialystok, Bialystok, POL

**Keywords:** zollinger-ellison syndrome, nuclear medicine, somatostatin, ulcer, zes, gastrinoma

## Abstract

Zollinger-Ellison syndrome (ZES) is a rare condition caused by gastrin-secreting neuroendocrine tumors known as gastrinomas. We present a case of hepatic ZES presenting as upper gastrointestinal (GI) bleeding in a 70-year-old female. Initial evaluation revealed the patient to be in severe sepsis with septic shock secondary to a urinary tract infection, and her hospitalization was complicated by hematemesis and melena in the setting of multiple duodenal ulcers. Subsequent investigations, including elevated gastrin levels and a somatostatin receptor scan, confirmed the diagnosis of gastrinoma.

## Introduction

Zollinger-Ellison syndrome (ZES) is caused by hypersecretion of gastrin by neuroendocrine tumors, known as gastrinomas, found most commonly in the stomach, duodenum, or pancreas [1]. Although ZES is commonly discussed as a cause of upper gastrointestinal (GI) bleeding in the literature, it is rarely seen in practice. Here, we present a case of hepatic ZES presenting as an upper GI bleed.

## Case presentation

A 70-year-old female with a history of cervical radiculopathy was brought to the emergency department following an unwitnessed fall at home. She was found by the paramedics to be hypotensive to 60/40 mmHg, hypothermic, and unresponsive to voice or painful stimuli. Upon arrival in the emergency department, her blood pressure had begun to stabilize with fluid resuscitation. She was tachycardic to 116 beats per minute, and her temperature was 96.7°F. She was unable to recall events around the fall but mentioned weakness and reduced oral intake for two days prior to admission. Lab results were significant for hemoglobin of 17.9 g/dL, white count of 19.2 k/mm3, creatinine of 2.99 mg/dL, calcium of 10.8 mg/dL, lactic acid of 7.6 mmol/L, creatine kinase of 340 IU/L, and troponin of 339 pg/mL. She was started on ceftriaxone for a suspected urinary tract infection per urinalysis and sent to the intensive care unit (ICU) for the management of severe sepsis. A CT from the head to the chest was remarkable only for an incidental 4 cm left thyroid mass. Thyroid-stimulating hormone (TSH) was low with normal free thyroxine (T4) and free triiodothyronine (T3).

**Figure 1:**
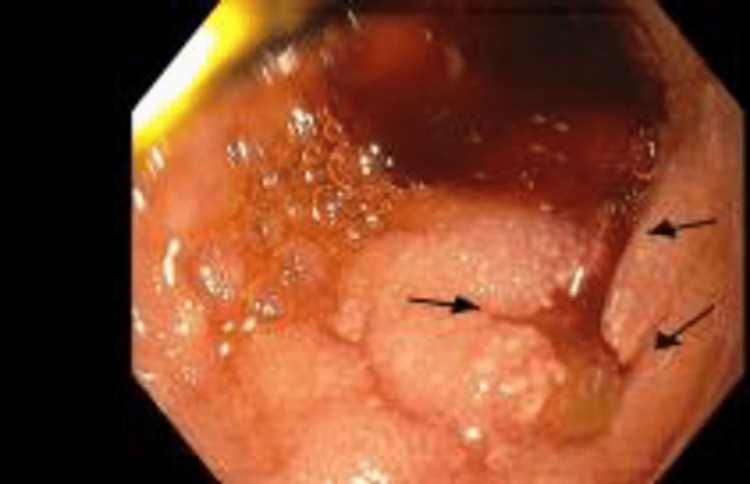
The first endoscopy showed a bleeding duodenal bulb ulcer.

While in the ICU, she experienced an episode of hematemesis and melena associated with a drop in hemoglobin to 10.6 g/dL. Gastroenterology was consulted. Upper endoscopy showed four oozing duodenal bulb ulcers with adherent clots in the duodenal bulb and first portion of the duodenum, with the largest measuring 12 mm (Figure 1).

**Figure 2:**
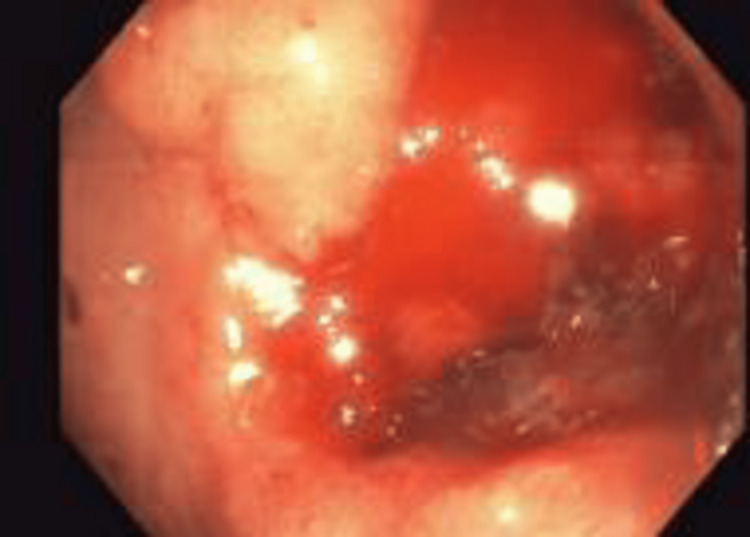
The second endoscopy showed an ulcer in the second part of the duodenum.

Three hemostatic clips were placed with cessation of bleeding post-procedure. Two days later, she was again found to be hypotensive and tachycardic with altered mentation. A repeat upper endoscopy identified additional ulcers in the second portion of the duodenum. Epinephrine was injected, and seven hemostatic clips were placed (Figure 2).

**Figure 3:**
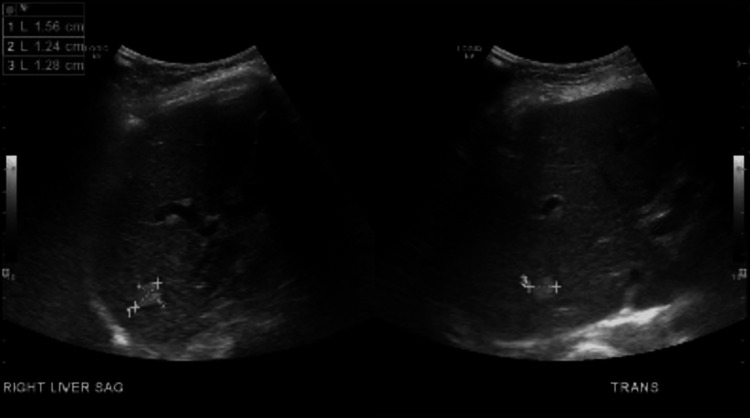
The ultrasonography of the liver showed a 1.6 cm hepatic lesion.

The ultrasound of the abdomen incidentally found a 1.6 cm right hepatic lobe lesion (Figure 3).

**Figure 4:**
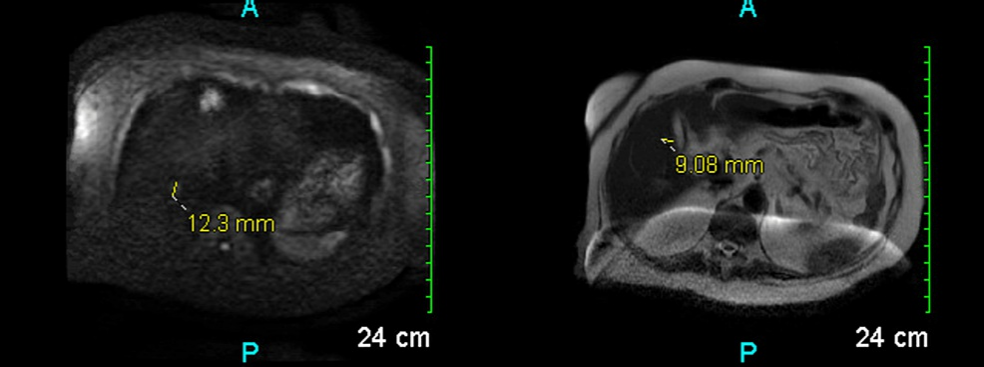
Magnetic resonance imaging revealed two echogenic foci in the liver.

Follow-up with an abdominal MRI revealed two small echogenic foci within the liver, one near the dome of the posterior segment of the right lobe and the second in the gallbladder fossa (Figure 4).

**Figure 5:**
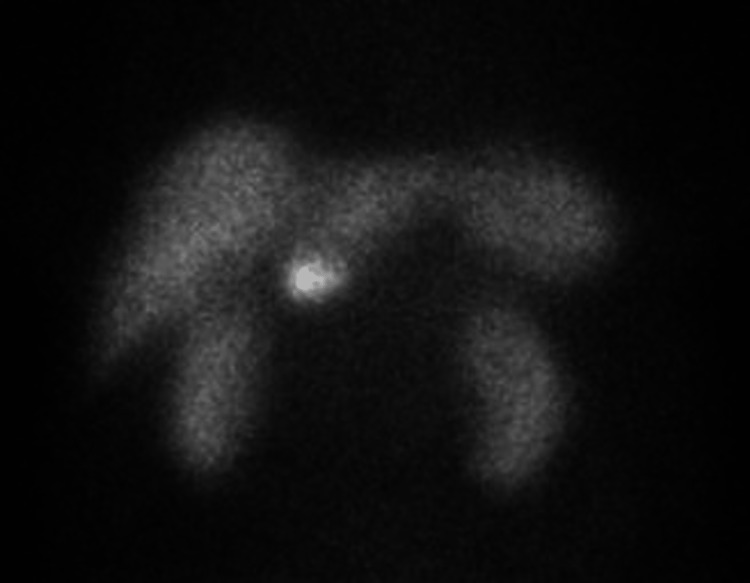
An indium-111 octreotide nuclear medicine scan showed a focus on increased activity within the liver.

Gastrin levels were ordered and found to be elevated to 6074 pg/mL (normal reference range: 0-180 pg/mL), raising suspicion for Zollinger-Ellison syndrome. An octreotide nuclear medicine scan was performed and showed a focus of abnormally increased activity within the right upper quadrant (Figure 5), confirming the diagnosis of gastrinoma.

Our patient was then started on a pantoprazole drip for 72 hours and later switched to a high-dose oral proton pump inhibitor (PPI). She refused any further invasive in-patient procedures and, hence, was continued on oral PPI to be followed as an outpatient for further workup. She was discharged to a skilled nursing facility for deconditioning with no further episodes of bleeding. She did not follow up with an outpatient upper endoscopy for an ultrasound-guided biopsy of the gastrinoma and confirmation of ulcer healing.

## Discussion

About 95% of gastrinomas arise in the gastrinoma triangle. The second and third portions of the duodenum, the pancreatic head, and the hepatoduodenal ligament act as its anatomic boundaries. Hepatic gastrin-secreting tumors are thus a rare finding, with less than 40 cases reported previously in the literature [1]. Regardless of location, all gastrinomas can manifest as ZES, presenting as either peptic ulcer disease, diarrhea, or gastroesophageal reflux disease that is often refractory to conventional management. Patients can also present with complications of gastric acid hypersecretion, including GI bleeding, strictures, or perforations [2]. Zollinger-Ellison syndrome should be suspected in individuals with multiple or refractory peptic ulcers, ulcers distal to the duodenal bulb, enlarged gastric folds, and a personal or family history of multiple endocrine neoplasia type 1 (MEN 1). About 80% of the cases are sporadic in nature, whereas the rest are associated with autosomal dominant syndrome MEN 1 [3,4]. In retrospect, our patient did not have any history to suspect ZES.

Due to its rarity, many gastrinoma cases go either unnoticed or are metastatic at the time of diagnosis. About 60% of gastrinomas are malignant, and metastasis is associated with high mortality [5,6]. 

Diagnostic workup begins with the measurement of fasting serum gastrin levels while off PPIs. Though very sensitive, values higher than five- to 10-fold of normal are non-specific, and thus a gastric pH level of two or lower is required for diagnosis. Appropriate rises in gastrin can be seen with the use of PPIs, pernicious anemia, and pan-gastritis *Helicobacter pylori* infection. Those can lead to gastrin levels up to 10 times the norm. On the other hand, ZES causes an inappropriate rise in gastrin due to the already-present hyperchlorhydria. Gastrin provocative tests such as secretin stimulation become necessary diagnostic methods for those with fasting gastrin levels below the 10-fold elevation mark. It works by stimulating gastrin release from gastrinoma cells while inhibiting its release from gastric G cells. When done off PPIs, a rise in serum gastrin of more than 120 pg/mL is typically considered positive. Another option would be to check the basal acid output, which is expected to be greater than 15 mEq per hour [5,7]. Localization using conventional imaging modalities like ultrasound, contrast-enhanced CT, and MRI can miss up to a third of patients with sporadic gastrinomas [3]. Nuclear imaging with somatostatin receptor scintigraphy improves localization accuracy. These receptors are expressed in the majority of well-differentiated neuroendocrine tumors. Positron emission tomography using radiolabeled somatostatin radiotracers like 111-indium pentetreotide or gallium-68 DOTATATE is typically utilized. Because of its greater sensitivity, gallium-68 DOTATATE is preferred over conventional somatostatin with 111-in pentetreotide where available [8]. In rare cases where other modalities are unable to pick up the lesion, we have to reserve for invasive testing to localize the tumor using either angiography or selective arterial stimulation and venous sampling with secretin injection. As a last resort, patients need to undergo laparotomy, as in some cases the tumor can only be localized by direct palpation, duodenal transillumination, or intraoperative ultrasound [4].

Tumor immunohistochemistry is used to confirm the diagnosis of gastrinoma, typically staining positive for chromogranin and synaptophysin and negative for alpha-fetoprotein, carcinoembryonic antigen, and cytokeratin-7 [9].

Zollinger-Ellison syndrome is treated to prevent further damage from hypergastrinemia, starting with high-dose PPIs. Mortality is largely dependent on the tumor being malignant or benign. Patients who are good candidates can undergo surgical resection, whereas those cases where the tumors are unresectable and have predominant hepatic metastasis can make use of embolization, selective internal radiation therapy, radiofrequency ablation, or cryoablation [10]. Survival rates are favorable following resection, though recurrences are frequent [11].

## Conclusions

Zollinger-Ellison syndrome is an exceedingly rare diagnosis frequently associated with sporadic gastrinomas, though some cases appear as part of the MEN 1 syndrome. It is an important diagnostic consideration for those with refractory upper gastrointestinal tract ulcers, especially when post-bulbar in location. Nuclear medicine is a useful diagnostic method given the low accuracy of serum gastrin levels. A correct diagnosis is of utmost importance given the malignant potential and poor prognosis.
